# A Text-Book of Practical Medicine Designed for the Use of Students and Practitioners of Medicine

**Published:** 1890-08

**Authors:** 


A TEXT-BOOK OF PRACTICAL MEDICINE DESIGNED FOR THE USE OF STUDENTS AND PRACTITIONERS OF MEDICINE. By ALFRED L. LOOMIS, M. D., LL. D, Professor of Pathology and Practical Medicine, in the Medical Department of the University of the City of New York; Visiting Physician to Bellevue Hospital, etc. Eighth edition; revised and enlarged with 215 illustrations; 8vo; pp. 1147. New York: William Wood & Co. 1889.

The fact that this book has reached an eighth edition in five years, sufficiently indicates its popularity—at any rate among the students of the University of the City of New York. Nevertheless, this does not make any less inexcusable its first appearance, since it is merely a reproduction of other men's ideas, fortified though it may be by a certain amount of experience on the part of the writer. The advisability of writing a book on the practice of medicine is questionable, when the author has nothing original to bring forward in the subject matter, even if the arrangement of the subject be new. However, this treatise is fairly well put together, and some points deserve especial mention. The introductory chapter on inflammation particularly is well worth perusal.

The body of the book is divided into six sections, the first of which is devoted to Diseases of the Respiratory Organs. It is a little surprising that in this edition, published in 1889, "all of which has undergone careful revision," no mention whatever is made of the local use of cocaine in acute coryza. A mode of treatment that has become so general, should at least be chronicled, even if only to be condemned by the writer.

After discussing the nature of acute lobar pneumonia at some length, our author concludes that it is "a general constitutional disease with local manifestations, undoubtedly depending upon a specific cause." In this he follows the teachings of Draper, Juergensen, and Flint. Is it not more in accordance with clinical observation to conclude that in acute lobar pneumonia, as in diphtheria, we have a disease originally local from which is developed a general septic fever? The statement that "there is no relation between the amount of lung involved and the intensity of the symptoms," is no proof whatever of the constitutional character of the disease; for no one, we suppose, would claim that acute follicular tonsillitis was a general disease with local manifestation; and still in that affection "there is no relation between the amount of tissue involved and the intensity of the symptoms." Another statement that "no second chill occurs when another lobe, part, or the other lung is attacked," is only partially true, for there is always at such time a great exacerbation of the fever — the second stage of a rigor. Moreover, the author makes no mention of a fact that goes far towards proving the local nature of the inflammation, viz., that the disease can be arrested at *any* stage in its progress, and that with the arrest of the inflammatory processes in the lung the general fever and other symptoms subside.

The recommendations for the treatment of pneumonia are good, except the objection to the use of carbonate of ammonia because, if given in sufficiently large doses to act as a stimulant, it irritates the stomach. Such gastric irritation may be prevented by giving the remedy in milk or mucilage of acacia. In our hands it has proved the most useful of drugs in this disease, because, in addition to its stimulating effects upon the heart, it has a very beneficial effect upon the pulmonary mucous membrane, thinning and loosening the fibrinous exudate.

One little paragraph on the etiology of phthisis presents so accurately the modern view, that it is worth quoting :

Although the bacillus is accepted as the sole *exciting* cause of phthisis, its omnipresence and the impossibility, under the present social conditions, of avoiding a more or less intimate contact with it, render the predisposing causes of its development, without which it is inactive, of paramount clinical importance. These causes are general and local. The most important general causes are an inherited or acquired feebleness of constitution, anti-hygienic influences, climate and soil. The local causes are inflammatory conditions of the pulmonary tissues.

In the section devoted to Diseases of the Digestive System, the diseases of the stomach are very poorly treated, the use of lavage as a means of diagnosis in the various forms of gastric indigestion not even being mentioned. It seems almost criminal to place in the hands of young practitioners, a text-book of medicine that handles the treatment of "summer complaint in children" in the manner in which this book does. The only good point mentioned is the use of calomel, and the author does not state the special indications for that drug, nor how long its use should be continued. It is little wonder that, with such teaching to fall back on, "the death-rate from this disease in New York City is twice that of any other city in the world." Neither the sterilization of milk, nor any substitute for milk as a food, is even mentioned. We trust that, for the sake of his patients, Dr. Loomis practises better than he preaches.

The other parts of this section deserve no especial mention in the way of either praise or condemnation. Section III., devoted to to Diseases of the Heart, Blood Vessels and Kidneys, surprises us by its superficiality and lack of originality, in a department where we might expect to find evidences of careful clinical and experimental research on the part of the author.

In the section on Acute General Diseases, diphtheria claims our attention. The morbid anatomy, etiology, clinical history, diagnosis and prognosis are all fairly well given, but when we come to the treatment, we are surprised to find in this book, which "has undergone careful revision" in 1889, no mention whatever made of the use of various digesting agents for the destruction of the membrane, nor of the operation of intubation, which has so largely taken the place of tracheotomy for the relief of laryngeal diphtheria. Of tracheotomy itself the merest mention is made, and the special after-treatment of the patient upon whom the operation has been performed, has been left entirely to the imagination. As the success of the operation of tracheotomy in diphtheria depends almost entirely upon the careful after-treatment, this omission, in addition to its numerous other faults and few virtues, should be enough to condemn this treatise entirely as a text-book for students and a reference book for young physicians.

It is unnecessary to point out the further shortcomings of this work, but there is not a section and scarcely an article that is not in need of "careful revision" by some one capable of making it.

The binding and letterpress are all that could be desired.

DeL. R.

